# Case Report: Endovascular treatment of spontaneous iliac vein rupture complicated with lower extremity deep vein thrombosis

**DOI:** 10.3389/fmed.2026.1876268

**Published:** 2026-06-17

**Authors:** Yun-Dong Chen, Qi Dong

**Affiliations:** 1 Department of Interventional Radiology, Nanjing Hospital of Chinese Medicine Affiliated to Nanjing University of Chinese Medicine, Nanjing, China; 2 Department of Otolaryngology Head and Neck Surgery, Affiliated Drum Tower Hospital of Nanjing University Medical School, Jiangsu Provincial Key Medical Discipline (Laboratory), Nanjing, China

**Keywords:** case report, covered stent implantation, deep vein thrombosis, endovascular treatment, spontaneous iliac vein rupture

## Abstract

**Background:**

Spontaneous iliac vein rupture (SIVR) is an extremely rare life-threatening vascular emergency with a 22% reported mortality. It predominantly affects middle-aged women, mostly involves the left iliac vein, and is frequently complicated by lower extremity deep vein thrombosis (DVT). Its nonspecific manifestations often lead to misdiagnosis and delayed treatment. This report presents a SIVR case with DVT and reviews relevant literature to inform clinical practice.

**Case presentation:**

The patient was a 60-year-old female admitted for sudden onset of lower abdominal pain and left lower extremity swelling after squatting, with stable hemodynamics. Abdominal computed tomography (CT) revealed pelvic hematoma, lower extremity ultrasound confirmed extensive left lower extremity DVT, and left external iliac vein rupture was diagnosed by venography. The patient underwent covered stent implantation combined with percutaneous mechanical thrombectomy (PMT) and postoperative catheter-directed thrombolysis (CDT). The rupture was successfully occluded, and the thrombus was completely removed. During the 1-year follow-up, the stent maintained favorable morphology and luminal patency, with no occurrence of post-thrombotic syndrome.

**Conclusion:**

SIVR predominantly occurs in middle-aged and elderly women, almost always involves the left iliac vein, and is frequently complicated with DVT. Venous compression associated with May-Thurner syndrome (MTS) is a critical predisposing factor. Venography is the key to definitive diagnosis. Endovascular treatment has shown favorable efficacy and safety in hemodynamically stable patients, and individualized anticoagulation strategies help improve long-term prognosis.

## 1 Introduction

Spontaneous iliac vein rupture (SIVR) is an extremely rare but life-threatening vascular emergency, with a reported mortality rate as high as 22%. (1, 2). A comprehensive literature search was performed in PubMed, EMbase, The Cochrane Library, Web of Science, and Scopus using the search strategy “(spontaneous) AND (iliac vein*) AND (rupture*)”, which identified a total of 66 English-language and 9 non-English-language publications documenting this disease entity. Just over 10 articles have reported the clinical application of endovascular therapy in the management of SIVR. More than 95% of SIVR cases involve the left iliac vein, the vast majority of patients are female with a mean age of around 60 years, and most cases are complicated with lower extremity deep vein thrombosis (DVT) (2–4). The clinical manifestations of SIVR are non-specific, including acute abdominal pain, hypovolemic shock and retroperitoneal hematoma, making it highly susceptible to misdiagnosis as ruptured abdominal aortic aneurysm, gynecological emergency or traumatic bleeding, resulting in a high rate of delayed diagnosis (5, 6). Therefore, it is critical to improve clinical awareness of this disease and standardize its diagnosis and treatment pathway. This paper reports the diagnosis and treatment course of a patient with left-sided SIVR complicated with DVT, and conducts a analysis of its etiology, diagnosis and treatment combined with literature review, to provide reference for clinical practice.

## 2 Case report

A 60-year-old female patient was admitted to our hospital with lower abdominal pain for 2 days and left lower extremity swelling for 1 day. The patient developed sudden distending pain in the lower abdomen during squatting 2 days prior, and was treated with anti-shock therapy for hemorrhagic shock at a local hospital, where abdominal CT showed pelvic hematoma. One day later, she developed secondary left lower extremity swelling, and ultrasound examination revealed left lower extremity DVT. An inferior vena cava (IVC) filter was implanted at the local hospital, and the patient was then transferred to our hospital.

The patient had no significant past medical history, and denied any history of trauma or surgery. Vital signs on admission were stable: body temperature 36.5 °C, pulse 78 beats/min, respiratory rate 20 breaths/min, blood pressure 125/68 mmHg, and oxygen saturation 98%. Physical examination showed a soft abdomen with mild tenderness in the lower abdomen, no rebound tenderness or muscle tension, and negative shifting dullness. The left lower extremity presented with obvious pitting edema, with normal skin color and temperature, and palpable dorsalis pedis artery pulse. Neurological examination revealed the patient was conscious and alert with fair mental status. Bilateral patellar tendon reflexes were intact, and Babinski, Hoffmann, and Kernig signs were negative bilaterally.

Laboratory examination results were as follows: hemoglobin 82 g/L (↓), red blood cell count 2.64 × 10^12^/L (↓), hematocrit 23.7% (↓), platelet count 109 × 10^9^/L (↓), prothrombin time (PT) 11.7 s, activated partial thromboplastin time (APTT) 26.9 s, fibrinogen 3.19 g/L, D-dimer 7.89 mg/L FEU (↑). Imaging examination: Abdominal CT revealed a localized pelvic hematoma that was ill-demarcated from the left iliac vein, with progression compared with the previous CT scan (Figure 1). Color Doppler ultrasound of bilateral lower extremity veins showed thrombosis in the left superficial femoral, deep femoral, popliteal and posterior tibial veins.

**Figure 1:**
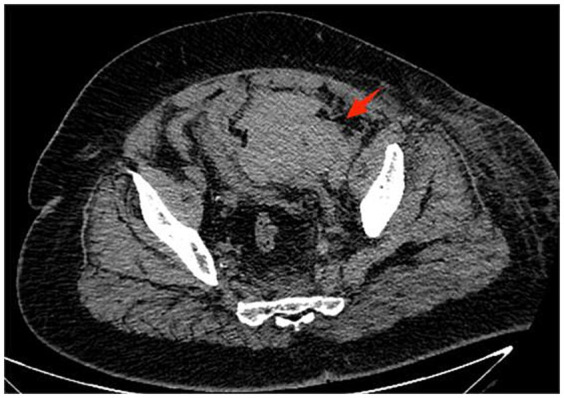
Abdominal CT reveals a large, mass-like hyperdense area in the pelvic cavity, poorly demarcated from the left iliac vein, suggestive of a pelvic hematoma.

Combined with the patient’s symptoms, signs, laboratory and imaging findings, left-sided SIVR complicated with DVT was highly suspected. After multidisciplinary discussion, emergency endovascular treatment was decided. Antegrade puncture of the left popliteal vein was performed under ultrasound guidance, and venography showed contrast extravasation at the distal end of the left external iliac vein (Figure 2). One 13 × 100 mm VIABAHN endoprosthesis (GORE, USA) was accurately implanted at the mid-distal left iliac vein rupture site, and repeat venography showed complete resolution of contrast extravasation. Subsequently, a 10F AcoStream thrombus aspiration system (Acotec, China) was advanced to repeatedly aspirate the left iliofemoral and popliteal veins, with a large amount of dark red fresh thrombus removed (Figure 3). Repeat venography showed restored blood flow and basically patent lumen of the left iliofemoral and popliteal veins, with a small amount of residual mural thrombus at the proximal iliac vein and the orifice of the femoral vein.

**Figure 2:**
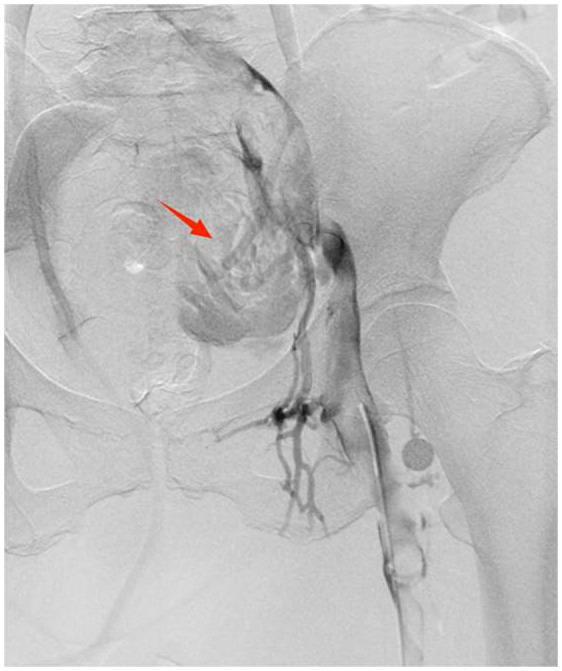
Venography demonstrated contrast extravasation from the distal left external iliac vein with concomitant left iliofemoral venous thrombosis.

**Figure 3:**
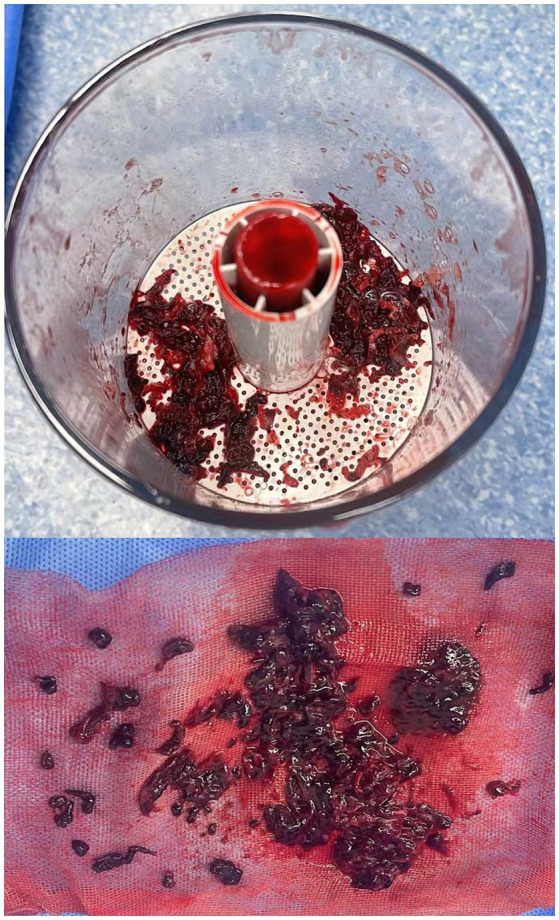
A large amount of fresh, dark-red thrombus was aspirated and retrieved from the left iliofemoral-popliteal vein using a 10-Fr peripheral thrombectomy aspiration catheter.

After the procedure, the sheath was retained, and the 20 cm Fountain infusion system (Merit, USA) was left in the left iliofemoral vein for continuous infusion of urokinase 200,000 U/day, with argatroban 40 mg/day infused through the catheter sheath. Coagulation function and hemoglobin levels were dynamically monitored. After 72 h of catheter-directed thrombolysis (CDT), repeat venography showed patent lumen of the left iliofemoral and popliteal veins with nearly complete thrombus clearance, and the patient’s left lower extremity swelling completely resolved. The IVC filter was then retrieved. Following discharge, the patient was maintained on standardized anticoagulant therapy with rivaroxaban according to the following regimen: 15 mg orally twice daily for the first 3 weeks, followed by 20 mg orally once daily from week 3 to month 3, and then 10 mg orally once daily from month 3 to month 6. No bridging antiplatelet therapy was administered. Regular follow-up was conducted for 1 year. Abdominal CT showed gradual absorption of the pelvic hematoma, with satisfactory patency and configuration of the left iliac vein stent (Figure 4). No in-stent restenosis or recurrent thrombosis was observed, and no clinical manifestations of post-thrombotic syndrome (PTS) occurred (see Table 1).

**Figure 4:**
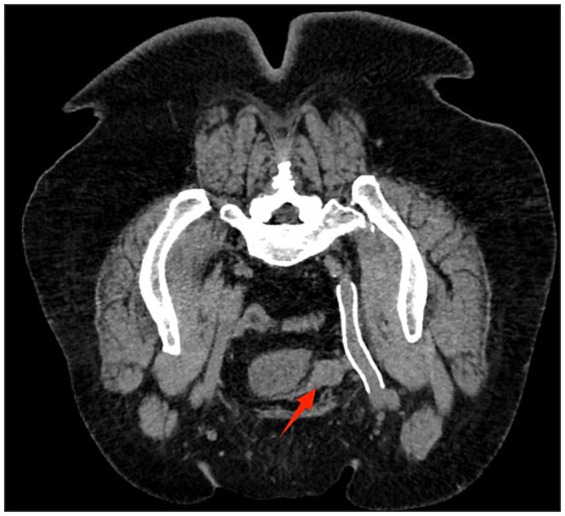
Follow-up abdominal CT at 6 months postoperatively demonstrated significant resolution of the pelvic hematoma, with the left iliac vein stent in good position and configuration.

**Table 1:** Timeline.

Time point	Clinical events
Day 0	Sudden-onset lower abdominal pain; pelvic hematoma identified on CT, diagnosed with hemorrhagic shock; emergent fluid resuscitation and blood transfusion
Day 1	Left lower extremity swelling; left lower extremity DVT confirmed on ultrasound; IVC filter placement at outside hospital, transferred to our institution
Day 2	Pelvic hematoma progression on follow-up CT; left iliac vein covered stent implantation, aspiration thrombectomy, and catheter-directed thrombolysis
Day 5	Near-complete thrombus resolution on venography after 72 h thrombolysis; complete resolution of left leg swelling; IVC filter retrieval
Day 11	Discharged on regular oral rivaroxaban for anticoagulation
3/6/12-Month Follow-up	Gradual resolution of pelvic hematoma; persistent patency of the left iliac vein stent

## 3 Discussion

### 3.1 Etiology and predisposing factors

The exact etiology of SIVR has not been fully elucidated. Currently, it is considered to be the combined result of mechanical factors [May-Thurner syndrome (MTS) and DVT], hormonal levels, inflammatory processes, and triggering factors. MTS refers to chronic obstruction of the left iliac vein outflow tract and venous hypertension caused by compression of the left common iliac vein by the right common iliac artery (7). However, collateral circulation formed in simple MTS can provide partial compensation, which is usually insufficient to cause rupture. When DVT occurs on this basis, the collateral circulation is acutely occluded. If intra-abdominal or venous pressure rises sharply at this time, the pressure cannot be effectively released, which may induce rupture (4). DVT is more likely to be the cause of iliac vein rupture, rather than the result of iliac vein compression by retroperitoneal hematoma. For MTS diagnosis, CTV and MRV achieve >95% sensitivity and specificity. Intravascular ultrasound (IVUS) is increasingly utilized intraoperatively to identify and localize iliac vein obstruction etiologies (spiculations, intimal lesions from chronic adherent thrombus), visualize dynamic arterial compression, evaluate treatment efficacy, and detect residual thrombus (8). The absence of intravascular ultrasound (IVUS) evaluation in this patient precluded definitive exclusion of underlying MTS. In this case, the sharp increase in intra-abdominal pressure caused by squatting may be the direct trigger of rupture, while DVT led to further obstruction of the outflow tract, which synergistically promoted venous wall rupture. Postmenopausal estrogen decline impairs the collagen and smooth muscle structure of the venous wall, leading to reduced venous compliance and increased fragility, which is an important reason for the high incidence of SIVR in middle-aged and elderly women (9). The 60-year-old female in this case is exactly under this hormonal background. Inflammatory processes such as venous thrombophlebitis can destroy the integrity of the venous wall, while luminal obstruction and local inflammation caused by DVT itself further increase the risk of rupture (4). Notably, DVT may play a double-edged sword role in SIVR: on the one hand, it increases venous pressure load, and on the other hand, it reduces bleeding by limiting flow or locally sealing the rupture.

The main triggering factors include coughing, defecation, vomiting, bending over, Valsalva maneuver, after IVC filter implantation, as well as obesity (especially WHO grade III obesity), malignancy, and inherited hypercoagulable state (10, 11).

### 3.2 Clinical manifestations and diagnosis

The clinical manifestations of SIVR are acute and non-specific. The typical presentation includes sudden persistent pain in the lower abdomen or lumbosacral region, hypotension, tachycardia, and severe cases may develop hemorrhagic shock, while lower extremity swelling is secondary to DVT (2). Systemic hypoperfusion secondary to retroperitoneal hemorrhage may precipitate acute kidney injury, while extensive iliofemoral venous thrombosis can progress to phlegmasia cerulea dolens (12).

Imaging examination is the key pathway for definitive diagnosis, and a multimodal imaging strategy is recommended. Contrast-enhanced CT can clearly show retroperitoneal hematoma and its relationship with the iliac vein, which is the first-choice screening method in the emergency setting, and helps to differentiate from ruptured abdominal aortic aneurysm. However, due to the compression of the hematoma, contrast leakage and vascular rupture are not easily detected on abdominopelvic CT (13, 14). Ultrasound has high sensitivity for DVT, but has limited value in locating iliac vein rupture. Venography is the gold standard for diagnosis, which can directly show the site of contrast extravasation. Although its invasiveness limits its universal application, it should be performed decisively when SIVR is highly suspected and CT cannot confirm the diagnosis.

In this case, the rupture of the external iliac vein was confirmed by emergency venography, which once again confirmed its core value in definitive diagnosis. For middle-aged and elderly women with unexplained retroperitoneal hematoma complicated with ipsilateral DVT, SIVR should be included in the primary differential diagnosis, and angiography should be given priority for evaluation.

### 3.3 Treatment strategies

There is no unified guideline for the treatment of SIVR. The management of SIVR requires a multidisciplinary approach, particularly in elderly patients who frequently present with multiple comorbidities. Coordinated efforts among the surgical, critical care, and nursing teams can significantly improve patient outcomes (15). Stratified decision-making should be made according to hemodynamic status and the risk of active bleeding, and minimally invasive strategies are gradually becoming the mainstream (3).

Conservative management is suitable for patients with stable hemodynamics and no evidence of active bleeding, with inpatient close monitoring, fluid resuscitation and early anticoagulation as the core. Anticoagulation initiated within 24 h of onset is safe in patients with stable condition after fluid resuscitation and blood transfusion (16). Sebastian et al. showed that in young, non-malignant patients with acute iliofemoral DVT, short-term anticoagulation (3–12 months) was non-inferior to long-term therapy (>12 months) in terms of safety and efficacy (17). However, anticoagulation alone may increase the risk of PTS, and for extensive iliofemoral DVT, active thrombus removal should be considered after hemostasis (18).

Open surgery, as the traditional treatment, mainly includes direct suture of the rupture or iliac vein ligation and repair. However, it is associated with major trauma, high complication and mortality rates, and is currently mainly applicable to patients with extremely unstable hemodynamics and no access to endovascular intervention. Some patients may also require laparotomy for decompression if there is evidence of compartment syndrome (19).

Endovascular treatment has been increasingly applied in the past 20 years, showing outstanding advantages of minimal invasiveness, rapid recovery and definite hemostasis. Covered stent implantation can directly occlude the rupture and relieve venous compression. If no appropriate covered stent is available, overlapping double bare stent technique can be used as an effective alternative (20). For iliac vein stenting, the use of stents with a diameter greater than 12 mm and length exceeding 80 mm minimizes the risk of stent migration. (21) For the thrombus burden complicated with DVT, percutaneous mechanical thrombectomy (PMT) has a comparable thrombus clearance rate to CDT, but with shorter treatment time, lower cost and radiation dose, especially suitable for patients with high bleeding risk (22). In addition, embolization with coils or tissue adhesive (NBCA) is also a safe and effective minimally invasive option for patients intolerant to surgery (6, 23).

The endovascular treatment adopted in this case fully embodies the above principles: covered stent achieved immediate hemostasis by occluding the rupture, large-bore aspiration catheter achieved rapid thrombus debulking, and 72-h postoperative thrombolysis removed residual thrombus, ultimately achieving favorable short-term and long-term outcomes. Postoperative anticoagulation consisted of oral rivaroxaban for 3 to 6 months to balance the risks of thrombotic recurrence and rebleeding (19).

### 3.4 Clinical implications

The endovascular treatment regimen of “covered stent occlusion + PMT + short-term CDT” adopted in this case avoided the trauma of open surgery and effectively prevented post-thrombotic syndrome. This case suggests that for elderly women with sudden abdominal pain, retroperitoneal hematoma complicated with ipsilateral DVT, SIVR should be included in the differential diagnosis as early as possible, and contrast-enhanced CT and venography should be completed preferentially. While the primary goal of SIVR management is hemostasis, restoration of venous outflow is essential for definitive treatment (24). A key advantage of endovascular therapy is that it allows simultaneous management of concomitant DVT, thereby improving long-term clinical outcomes.

## 4 Conclusion

SIVR is a rare but life-threatening vascular emergency, which mainly occurs in middle-aged and elderly women, predominantly affects the left iliac vein, and is often complicated with DVT. MTS-related venous compression is the most frequently reported predisposing factor for SIVR (not definitively confirmed in this case due to lack of IVUS), and DVT-induced collateral occlusion plus acute venous pressure elevation are the core pathogenic triggers. Contrast-enhanced CT is the first-choice screening method in the emergency setting, and venography is the gold standard for definitive diagnosis. For treatment, stratification should be made according to hemodynamic status. Endovascular treatment has shown excellent efficacy and safety. Individualized postoperative anticoagulation management is the key to improving long-term outcomes.

## Data Availability

The raw data supporting the conclusions of this article will be made available by the authors, without undue reservation.
